# The Chemometric Evaluation of the Factors Influencing Cloud Point Extraction for Fluoroquinolones

**DOI:** 10.3390/pharmaceutics15061774

**Published:** 2023-06-20

**Authors:** Aleksandra Michałowska, Olga Kupczyk, Andrzej Czyrski

**Affiliations:** Department of Physical Pharmacy and Pharmacokinetics, Poznań University of Medical Sciences, Rokietnicka 3 Street, 60-806 Poznań, Poland

**Keywords:** central composite design, ciprofloxacin, green chemistry, HPLC, levofloxacin, moxifloxacin, optimization, recovery, sample pre-conditioning

## Abstract

This study aimed to analyze the factors that impact the cloud point extraction of ciprofloxacin, levofloxacin, and moxifloxacin. The following independent variables were analyzed: Triton X-114 concentration, NaCl concentration, pH, and incubation temperature. The dependent variable studied was recovery. A central composite design model was used. The applied quantitation method was HPLC. The method was validated for linearity, precision, and accuracy. The results underwent ANOVA^®^ analysis. The polynomial equations were generated for each analyte. The response surface methodology graphs visualized them. The analysis showed that the factor most affecting the recovery of levofloxacin is the concentration of Triton X-114, while the recovery of ciprofloxacin and moxifloxacin is most affected by pH value. However, the concentration of Triton X-114 also plays an important role. The optimization resulted in the following recoveries: for ciprofloxacin, 60%; for levofloxacin, 75%; and for moxifloxacin, 84%, which are identical to those estimated with regression equations—59%, 74% and 81% for ciprofloxacin, levofloxacin, and moxifloxacin, respectively. The research confirms the validity of using the model to analyze factors affecting the recovery of the analyzed compounds. The model allows for a thorough analysis of variables and their optimization.

## 1. Introduction

With the rapid development of green chemistry, whose premise is to use resources responsibly and reduce the generation of pollutants, new solutions and alternatives to existing analytical methods are being sought. These often use organic solvents that might harm both the researcher and the environment. One alternative method is cloud point extraction (CPE), which uses non-toxic surfactants instead of organic solvents. 

CPE was initially used only for the preconcentration of metals in the form of hydrophobic complexes. Over time, the potential it represented was noticed, and it began to be used more often, for example, as the first stage of isolation in protein purification [1]. It is also a safe method for the environment and the worker due to the limited use of harmful organic solvents. In addition, it is relatively cheap, fast, and universal and might be used to extract temperature-sensitive substances. In addition, the wide variety of surfactants with different cloud point temperatures and critical micelle concentrations makes it possible to adjust the reaction conditions for a specific analyte [1,2,3].

The CPE method is a form of modified liquid-liquid extraction (LLE). The basis of the method is that at low concentrations, below the critical micellar concentration (CMC), the surfactant takes the form of a homogeneous isotropic liquid, separated into two isotropic phases above the cloud point temperature. A turbid micellar phase rich in surfactant and an aqueous phase with a surfactant concentration close to CMC are formed. Above the CMC, surfactant monomers spontaneously aggregate into micelles of various sizes and shapes, depending on the type of surfactant [1]. The formation of the two layers may be enhanced by adding salt or changing the temperature. Regardless of the type of surfactant, when forming micelles, its molecules turn their hydrocarbon, hydrophobic tails towards the center, creating a non-polar core. Inside, it accumulates hydrophobic and covalent molecules present in a given aqueous solution [1,2,3,4].

CPE is widely used in the pretreatment of aqueous samples. It was applied in the determination of the following drugs in human plasma: amitriptyline and fluoxetine [5], venlafaxine [6], meloxicam [7], and bisoprolol [8]. Vitamins A, E, and K were determined in aqueous solutions, plasma, and urine [4]. Fluoroquinolones were determined in the environmental water samples [9], plasma, and urine [10]. CPE was also applied to determine cetylpyridinium chloride in the pharmaceutical formulations [11].

Reducing the use of materials is also part of process optimization through the design of experiments. Its application allows a significant reduction in the consumption of reagents needed to develop an analytical method. An additional advantage is the ability to analyze several variables simultaneously and their interactions. It gives a complete picture of how maximum results can be achieved by changing the conditions. Analyzing the influence of factors on the course of the process allows for a thorough understanding of the relationships in the system and the optimization of the process.

In this study, CPE was optimized for ciprofloxacin (CIPRO), levofloxacin (LEVO), and moxifloxacin (MOXI), which are representatives of the second, third, and fourth generation fluoroquinolones, respectively. The extracted agent was Triton X-114 (TX-114). The optimized parameters will be the concentration of TX-114 and NaCl, pH, and temperature. The dependent variable analyzed will be recovery. The applied technique will be optimized with a circumscribed central composite design (CCD). The ANOVA^®^ will indicate the most significant independent variables analyzed in single and their interactions. In an era of increasing prevalence of ecological approaches, including quantitative analysis, combining the abovementioned methods (CPE and design of experiments) increases efficiency while minimizing reagent consumption.

## 2. Materials and Methods

### 2.1. Reagents and Materials

CIPRO, LEVO, TX-114, triethylamine (TEA), sodium monophosphate, and orthophosphoric acid were purchased by Sigma Aldrich (Steiheim, Germany). MOXI was purchased by Santa Cruz Biotechnology (Dallas, TX, USA). Acetonitrile (ACN), methanol (MeOH), and potassium chloride were purchased by Merck (Darmstadt, Germany). All reagents were HPLC-grade. Sodium chloride, sodium hydroxide, boric acid, and acetic acid were purchased by Avantor Performance Materials (Gliwice, Poland). The HPLC analysis was done on LiChroCART^®^ 250-4, HPLC-Cartridge, and LiChrospher^®^ 100 RP-18 (5 μm) (Merck, Darmstadt, Germany) with a LiChroCART^®^ guard column (4-4, LiChrospher^®^ 100 RP-18 (5 μm)).

### 2.2. Solutions

The standard stock solutions of CIPRO, LEVO, and MOXI were prepared by dissolving a proper amount of analyte in the volumetric flask to reach the final concentration of 1 mg/mL of the analytes.

The working solutions of the analytes (100 mg/L for LEVO and MOXI, 60 mg/L for MOXI, 50 mg/L for CIPRO, and 50 and 20 mg/L for LEVO) were prepared by diluting the proper volume of standard stock solution in the volumetric flask.

Phosphate-buffered saline (PBS) was prepared by the dissolution of 0.8 g of NaCl, 0.2 g of KCl, 0.144 g of Na_2_HPO_4,_ and 0.024 g of KH_2_PO_4_ in water in a volumetric flask of 100 mL. The pH of 7.4 was shifted with phosphoric acid.

Britton-Robinson Buffer (BRB) is universal for the pH range of 2–12. It was prepared by mixing the proper amounts of boric acid, phosphoric acid, and acetic acid in the volumetric flask to reach the final concentrations for the analytes of 0.04 M. The final volume of the buffer was 250 mL. The titration achieved the desired pH with 0.2 M NaOH.

### 2.3. The Design Matrix and Statistical Analysis

The circumscribed CCD was applied in the analysis [12]. The star points were denoted as |α| = 2, and the remaining levels were −1, 0, 1, for four analyzed factors (independent variables): the concentration of TX-114 [%], the concentration of NaCl [%], pH, and incubation temperature. The response (dependent variable) was the recovery of the analyte. The design is presented in Table 1. The coded values are listed in Table 2. The method for the codification of the values was described by Bezerra et al. [13]. A second-order polynomial equation was fitted to correlate each independent variable to the response. The polynomial equations are presented in Section 3.1. They were applied for the calculation of theoretical values of recovery for each analyte. The non-significant parameters are not included. The equations with all variables and interactions are presented in the supplementary information.

The regression coefficients (R^2^ and R^2^_adj_) were calculated with analysis of variance (ANOVA^®^). The applied software was Statistica 13.3 (TIBCO Software Inc., Palo Alto, CA, USA). It was also applied in the statistical analysis. The statistical analysis comprised the analysis of the dependence of the recovery on the analyte’s concentration. The Shapiro-Wilk test was applied to check the normal distribution of the data. The Student’s *t*-test was applied to check whether the recovery depends on the concentration of each analyte. 

### 2.4. The Extraction Procedure and Quantitative Analysis

The CPE was optimized on PBS to reduce the use of plasma. Its osmolality and the concentration of the ions are similar to those in the human body, which is a substitute for plasma in the analysis [14]. TX-114 was used due to its low cloud point temperature of 23 °C [2].

The sample was prepared according to the following procedure: to 300 µL of PBS was added 30 µL solution of the analyte (MOXI or LEVO c = 100 mg/L for both analytes, for CIPRO c = 50 mg/L), 30 µL of internal standard (MOXI c = 20 mg/L for LEVO, LEVO c = 60 mg/L for MOXI, and LEVO c = 50 mg/L for CIPRO), and 300 µL of the buffer. TX-114, NaCl, buffer pH, and temperature levels are listed in Table 1 (the design matrix) and Table 2 (the coded levels of factors). The volume of the sample was constant—910 µL. Each experiment was repeated twice.

The samples underwent rotator stirring for 5 min. The prepared mixtures were incubated for 20 min. at the temperature defined for each experiment in Table 1. After incubation, the samples were centrifuged at 12,000× *g*. The water-rich phase was removed. The surfactant-rich phase was analyzed—it was dissolved in 200 µL of the mobile phase. The sample was injected into a chromatographic column. The volume of the injection was 20 µL. Two sets of samples were prepared to evaluate the recovery. In the first one, the analyte was extracted according to the procedure described above. In the second one, it was added before injection into the HPLC system—the analyte was dissolved in the mobile phase to obtain the same concentration equal to the theoretical concentration in the sample after dissolution in the mobile phase. To maintain the constant volume for the extraction procedure for the second series, 30 µL of methanol was added instead of the analyte solution. For the optimized conditions, the recovery was tested for the following concentrations: 1 and 8 mg/L. There were five repeats for each analyte and each concentration. Each analyte’s results underwent statistical analysis to check whether the recovery depended on the concentration.

The quantitative analysis was conducted with the validated HPLC method [15]. The chromatograms are presented in Supplementary Information (Figures S1–S3).

### 2.5. Validation of the HPLC Method

The methods were validated according to ICH criteria for precision, accuracy, and linearity [16]. The concentration range for the calibration curve was 0.5–10.0 mg/L for CIPRO and 0.2–10 mg/L for LEVO and MOXI. The mean calibration curve was calculated as the mean of five curves.

The precision (relative standard deviation—%*RSD*) was calculated according to the following formula:

$$\%RSD=\frac{SD}{\bar{X}}\times 100\%$$

where: *SD*—standard deviation, $\bar{X}$—mean value.

Accuracy was calculated with the following equation:

$$Accuracy=\frac{\left|c_{t}-c_{d}\right|}{c_{t}}\times 100\%$$

where: *c_t_*—theoretical concentration; *c_d_*—determined concentration.

The measurements were performed within a day (intraday) and on different days (interday). They were performed for the following concentrations: high: 8 mg/L (high—for all analytes); medium: 3 mg/L (for LEVO and MOXI) or 5 mg/L (for CIPRO); low: 1 mg/L (for CIPRO) or 0.5 mg/L (for LEVO and MOXI); and for LLOQ (lower limit of quantitation, the lowest concentration for the calibration curve): 0.5 mg/L (for CIPRO) or 0.2 mg/L (for LEVO and MOXI), for five repetitions for each condition and each analyte. The interday measurements were done for five days. The samples for calibration curves were prepared under optimized conditions for CPE extraction, characteristic of each analyte. They are listed in Section 4.5 according to the procedure described in Section 2.4.

## 3. Results

### 3.1. The Analysis of the Experiments

The statistical analysis confirmed the suitability of the applied chemometric model. The R^2^ and R^2^_adj_ values for CIPRO were 0.9938 and 0.9866, respectively. The values of R^2^ and R^2^_adj_ for LEVO were 0.9880 and 0.9739, respectively. In the case of MOXI, the R^2^ and R^2^_adj_ values were 0.9913 and 0.9813, respectively. The lack of fit was not statistically significant in all cases, and the differences between R^2^ and R^2^_adj_ did not exceed 0.015 for all analytes [12]. The analyzed independent variables were significant for the model, as confirmed by the high values of R^2^_adjusted_ for all analytes. The results of the ANOVA^®^ analysis are listed in Table 3, Table 4 and Table 5. The results of the experiments are in supplementary information (Tables S1–S3).

The polynomial equations described the recovery of the analyte. Below are the equations after removing the non-significant variables:
Recovery_CIPRO_ = 27.358 + 2.843 × TX-114 − 3.290 × (TX-114)^2^ − 1.389 × NaCl + 1.346 × (NaCl)^2^ − 7.077 × pH − 1.278 ×temperature − 1.460 × TX-114 × NaCl + 1.564 × TX-114 × pH + 1.377 × TX-114 × temperature + 0.727 × NaCl × pH + 1.027 × pH × temperature
Recovery_LEVO_ = 31.037 + 9.235 × TX-114 − 1.253 × (TX-114)^2^ − 4.005 × NaCl + 1.632 × (NaCl)^2^ − 3.451 × pH + 1.286 × pH^2^ − 2.570 × temperature + 2.209 × (temperature)^2^ + 1.349 × TX-114 × NaCl − 5.129 × NaCl × pH − 3.467 × pH × temperature 
Recovery_MOXI_ = 41.088 + 6.405 × TX-114 − 2.160 × (TX-114)^2^ + 0.861 × NaCl − 1.960 × (NaCl)^2^ − 11.154 × pH − 1.297 × temperature − 2.238 × TX-114 × pH + 1.123 × TX-114 × temperature − 1.801 × NaCl × pH + 1.295 × pH ×temperature

### 3.2. The Validation Parameters

The validation of HPLC analysis also confirmed the suitability of the method. The inter- and intraday precision and accuracy did not exceed 8%. The method was linear within the analyzed concentration ranges. The correlation coefficient exceeded 0.9995 for all analyzed compounds: the calibration curves were described by the simple linear model. The parameters for validation are presented in Table 6, Table 7 and Table 8.

## 4. Discussion

### 4.1. The Optimization of TX-114 Concentration

In CPE, the micelles of surfactant are the extracting agents. Their function is analogous to the immiscible organic layer in LLE. In our study, the analyzed concentration range of surfactant was 1.5–9.0%. Kojro et al. [2] suggest that surfactant concentrations should be within 1–9%. The lowest concentration in our study was 1.5%. In the lower concentration, the rich micelle layer did not separate. The increase in the concentration of the surfactant increases the recovery. However, too high a concentration may result in dilution of the analyte [2]. On the other hand, too low a surfactant concentration decreases accuracy and repeatability [1].

**Figure 1:**
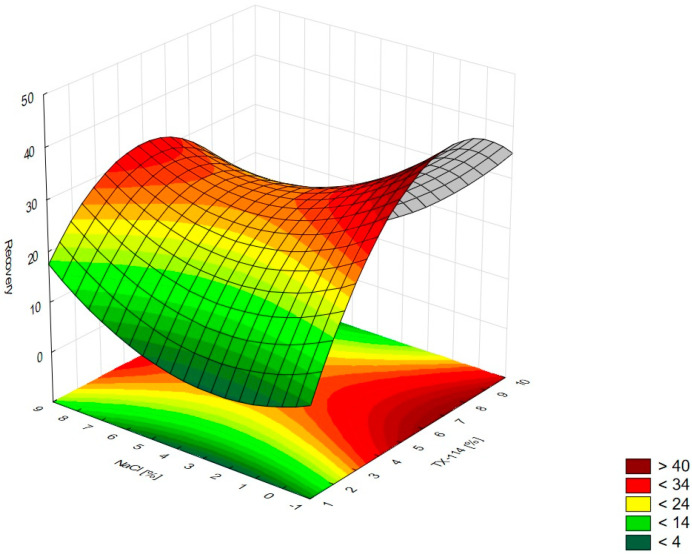
The RSM diagram for recovery of CIPRO when analyzing concentrations of TX-114 and NaCl.

**Figure 2:**
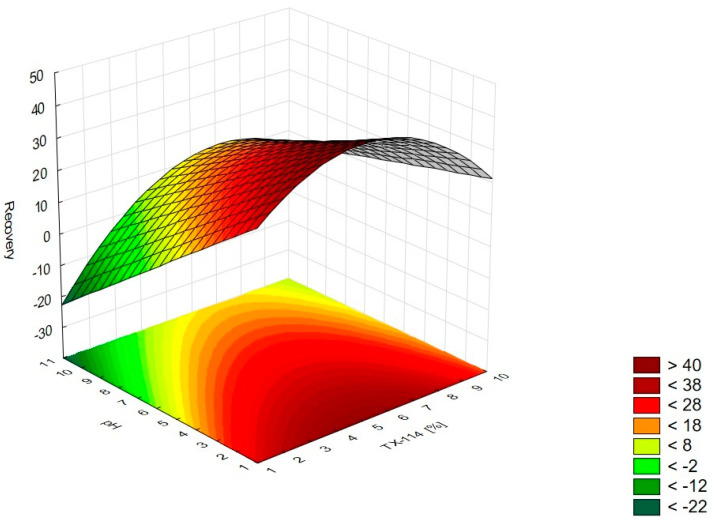
The RSM diagram for recovery of CIPRO when analyzing concentrations of TX-114 and pH.

**Figure 3:**
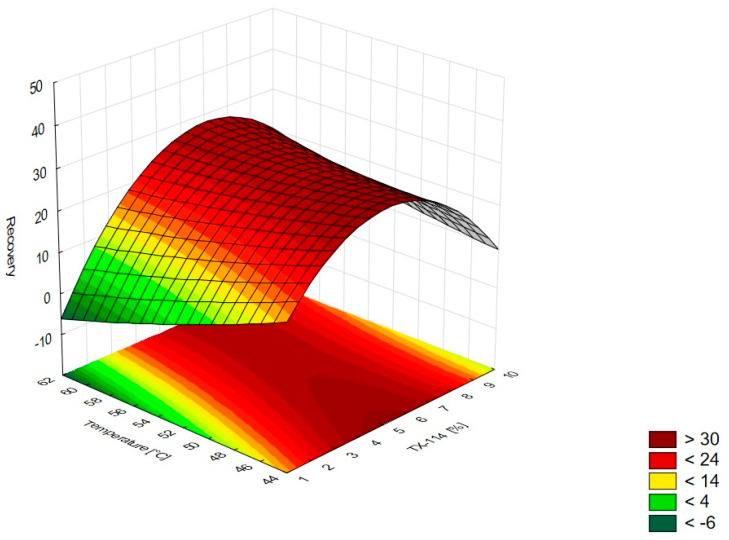
The RSM diagram for recovery of CIPRO when analyzing concentrations of TX-114 and temperature.

**Figure 4:**
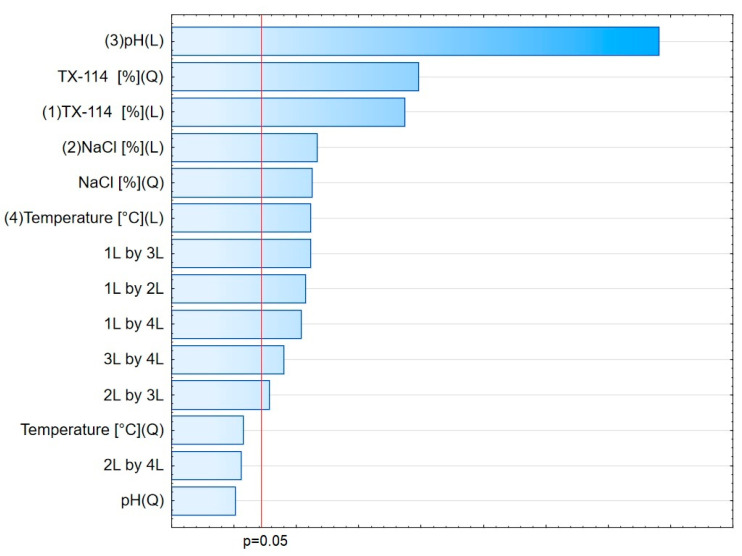
The Pareto chart for CIPRO.

In Figure 1, the RSM diagram takes a saddle shape, which indicates that there is no distinct maximum. The highest recovery of CIPRO is observed for the concentration of TX-114% 5–7% without NaCl addition. The addition of neutral salt (up to ca. 4%) results in a decrease in recovery regardless of the surfactant concentration. When the concentration of salt is higher than 5%, the recovery increases—it is observed for a high concentration of NaCl (ca. 9%) and TX-114 concentrations of 3–6%. However, the observed values are lower in this case. When pH is analyzed with TX-114 concentration (Figure 2), the highest recovery is observed for the concentration of surfactant (3–7%) at low pH values. The increase in pH leads to a decrease in recovery, regardless of the TX-114 concentration. For this range of surfactant concentration, the impact of temperature is insignificant, and the plateau is observed in Figure 3. For lower TX-114 concentrations (up to 3%), the impact is noticeable. The surfactant concentration, besides pH, is one of the most significant factors impacting the recovery of CIPRO (Figure 4).

**Figure 5:**
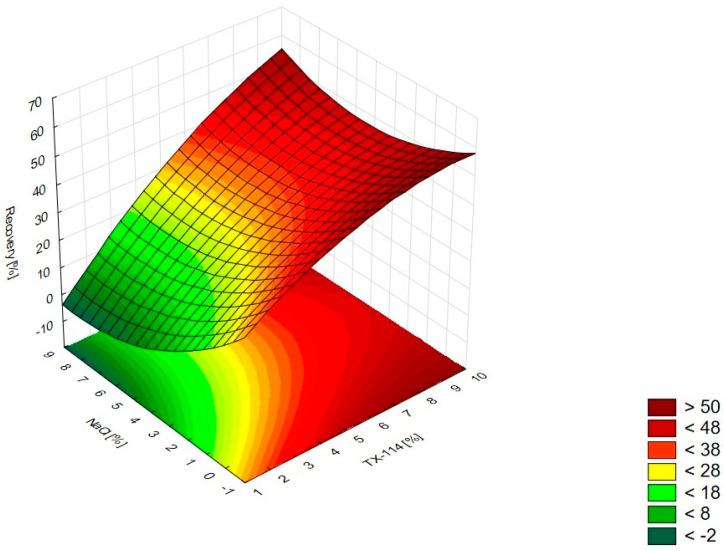
The RSM diagram for recovery of LEVO when analyzing concentrations of TX-114 and NaCl.

**Figure 6:**
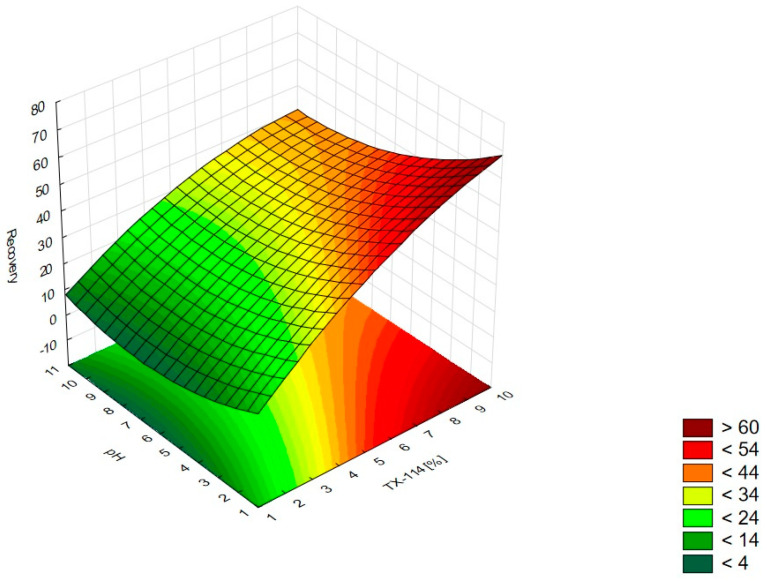
The RSM diagram for recovery of LEVO when analyzing concentrations of TX-114 and pH.

**Figure 7:**
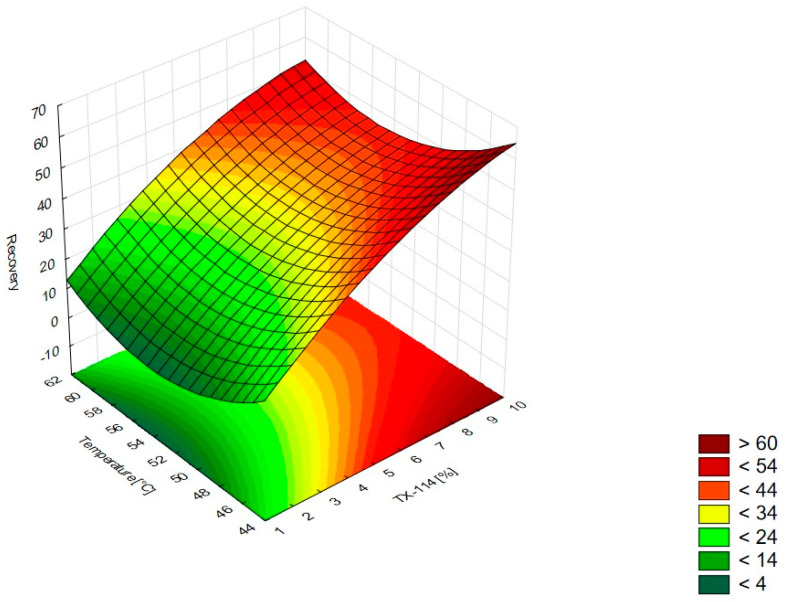
The RSM diagram for recovery of LEVO when analyzing concentrations of TX-114 and temperature.

**Figure 8:**
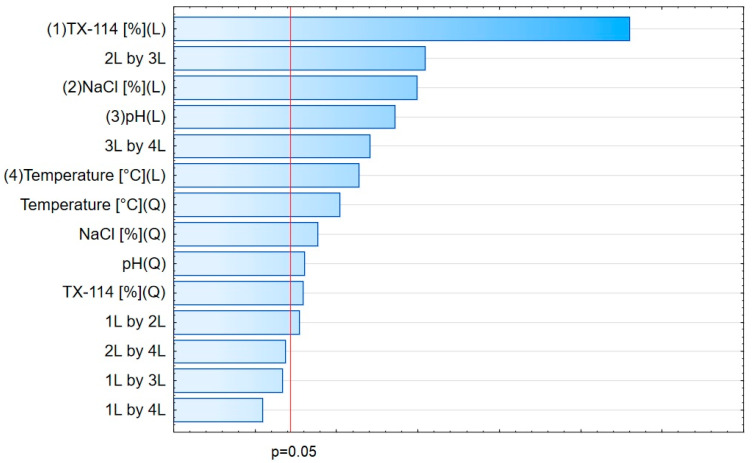
The Pareto chart for LEVO.

The analysis of RSM diagrams indicates that with the increase in TX-114 concentration, the recovery of LEVO increases regardless of NaCl concentration, pH, and temperature (Figure 5, Figure 6 and Figure 7). A significant increase in recovery is observed for concentrations 1.5–6%. The maximum is reached at a concentration of 9%. For higher surfactant concentrations, the impact of salt concentration is significant. The most detectable growth is observed when an increase in TX-114 is combined with a high salt concentration, and the recovery is highest for high concentrations of both TX-114 and NaCl (Figure 5). According to the Pareto chart, the concentration of TX-114 impacts LEVO’s recovery the most (Figure 8). The other significant factor is the concentration of NaCl when analyzed in single and in interaction with pH. The distinct maximum value is not observed for the concentration of TX-114. The recovery of LEVO increases when a high value of TX-114 is combined with a low pH, and the maximum is noted when a high concentration of surfactant is associated with the lowest pH from the analyzed range (Figure 6).

**Figure 9:**
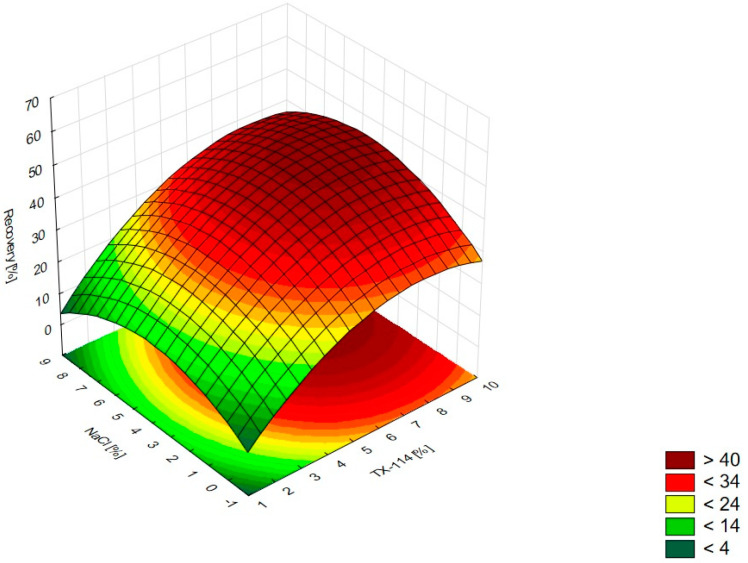
The RSM diagram for MOXI recovery when analyzing the concentration of TX-114 and NaCl.

**Figure 10:**
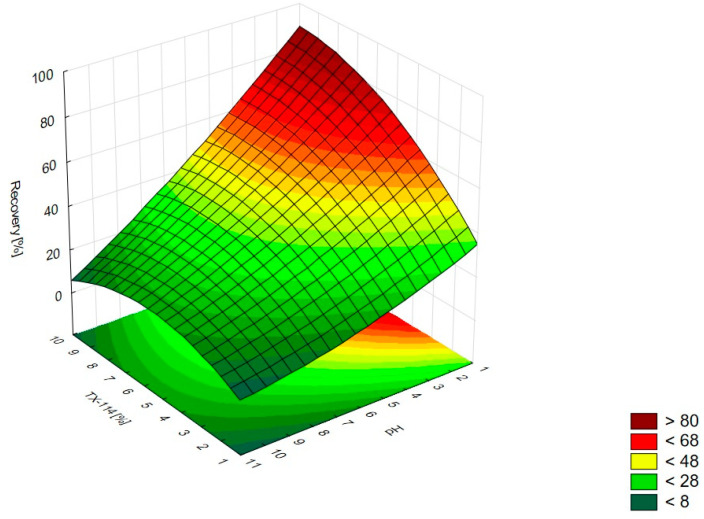
The RSM diagram for recovery of MOXI when analyzing concentrations of TX-114 and pH.

**Figure 11:**
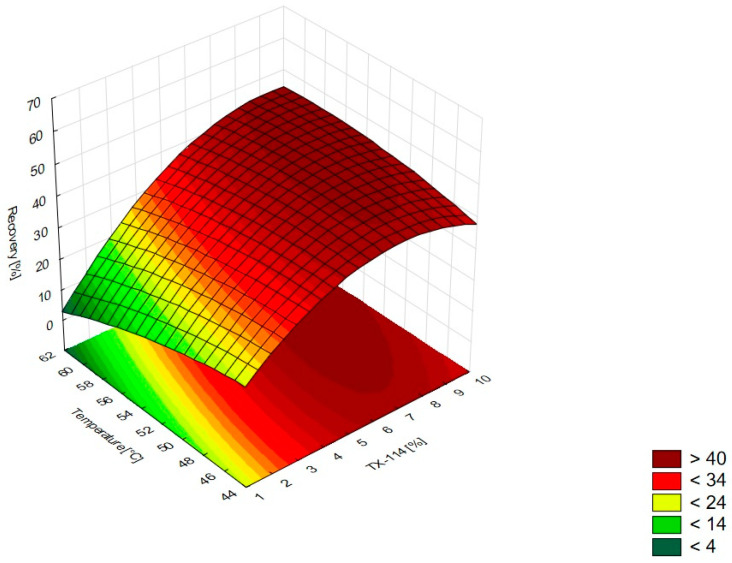
The RSM diagram for recovery of MOXI when analyzing concentration of TX-114 and temperature.

**Figure 12:**
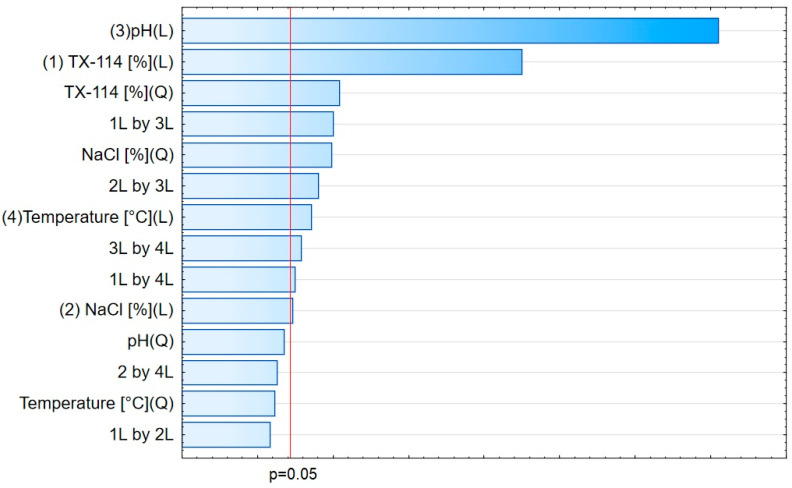
The Pareto chart for MOXI.

For MOXI (Figure 9, Figure 10 and Figure 11), the distinct maximum is observed in the RSM diagram (Figure 9) when the concentration of TX-114 is analyzed with NaCl concentration. In this case, a high recovery is observed for high concentrations of both a surfactant and salt. The highest recovery values are observed for TX-114 concentrations of 7–9% and NaCl concentrations of 3–6%. As in the case of LEVO, the highest recovery of MOXI is observed with a high concentration of TX-114 and low pH values. The increase in pH reduces the recovery regardless of the TX-114 concentration (Figure 10). In the case of MOXI, the combination of lower TX-114 concentrations and a higher temperature in the analyzed range resulted in a slight decrease in recovery. However, it is not a significant decrease (Figure 11). Contrary to LEVO, the concentration of TX-114 is not the most significant factor that has an impact on recovery—the most significant is pH (Figure 12).

### 4.2. The Optimization of NaCl Concentration

**Figure 13:**
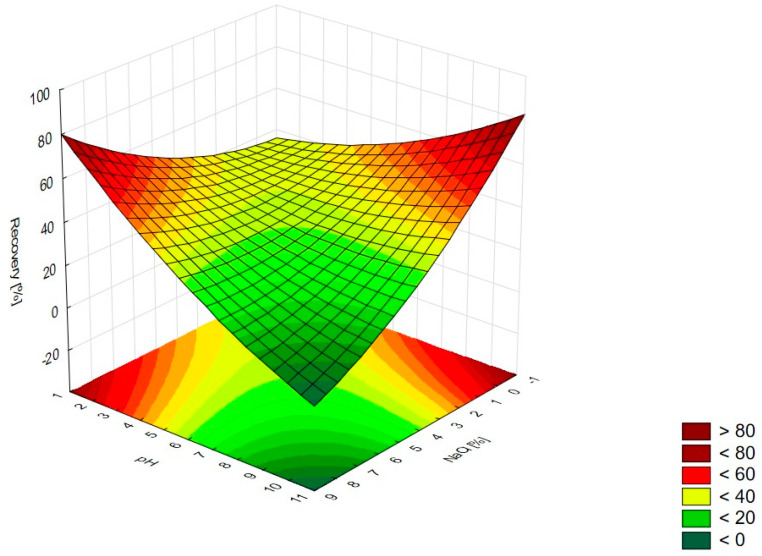
The RSM diagram for recovery of LEVO when analyzing concentration of NaCl and pH.

**Figure 14:**
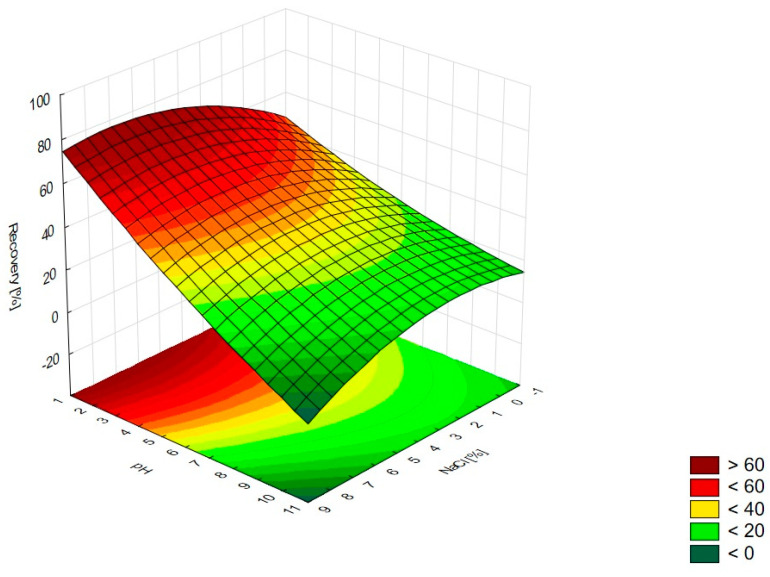
The RSM diagram for recovery of MOXI when analyzing concentration of NaCl and pH.

**Figure 15:**
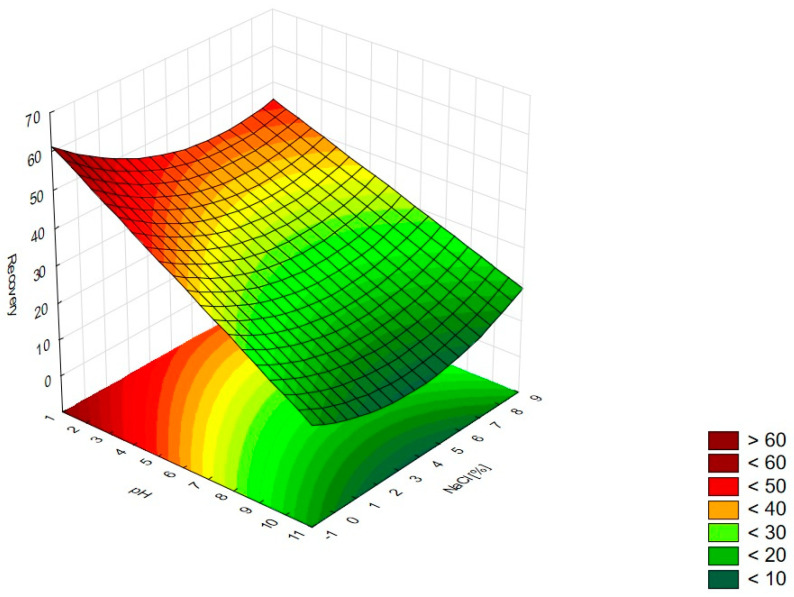
The RSM diagram for recovery of CIPRO when analyzing concentration of NaCl and pH.

The other factor that has an impact on recovery in the CPE is the ion force. The addition of neutral salts (NaCl in this study) impacts CMC—for nonionic surfactants it decreases upon adding NaCl. It also causes an increase of the number of micelles and their sizes. It leads to a reduction in the amount of water that is accessible for the dissolution of the analyte. The addition of neutral electrolytes increases recovery, especially for polar substances. The analysis of the salt concentration applied in CPE showed that it should be within 0.8–6.0% [1,2,4]. The analysis of the RSM diagrams for LEVO (Figure 5 and Figure 13) showed a high recovery for NaCl concentrations of ca. 4% and higher. It should be associated with a TX-114 concentration of at least 4%. MOXI has the highest recovery for salt concentrations of 3–6% (Figure 9 and Figure 14). For LEVO and MOXI, the decrease in pH combined with the reported concentrations of NaCl increased recovery. These findings follow the observations of Wu et al. [10], who indicated that the optimal value of NaCl concentration is 6% for gatifloxacin and ofloxacin. Kojro et al. also reported that the most commonly used concentrations of neutral salt are 4–6% [2]. For CIPRO, the maximum value of recovery is observed for low NaCl concentrations, which makes the addition of the salts not necessary, but the saddle shape of the RSM diagram shows that the increase in recovery is also noted for high concentrations of NaCl (6–8%). However, in this case, the recovery is lower (Figure 1 and Figure 15). The recovery increases for low pH values. The shift of pH towards higher values causes a decrease in CIPRO recovery regardless of the applied NaCl concentration.

### 4.3. The Optimization of pH

The recovery of the analyzed substances strongly depends on the pH applied. It impacts the ionization of the analyte in the micelle-rich layer. The most preferable is the neutral form [1,2]. On the other hand, Xia et al. [9] conducted the CPE with sodium dodecyl sulfate for norfloxacin, ciprofloxacin, sarafloxacin, and gatifloxacin in extreme acid pH (at 12 M hydrochloric acid). The addition of hydrochloric acid promoted phase separation and reduced the volume of the surfactant-rich phase. It resulted from the diminution of water content in the surfactant-rich phase. In the present study, a similar trend is observed—the highest recoveries are observed at the lowest pH from the analyzed range, i.e., at 2.0 for CIPRO, LEVO, and MOXI (Figure 2 and Figure 15, Figure 6 and Figure 13, and Figure 10 and Figure 14, respectively). The analysis of RSM diagrams showed that for all analytes, the increase in pH results in a decrease in recovery. The decline in recovery is noted when pH grows in all analytes, and it is augmented for low surfactant concentrations. It implies that a high surfactant concentration should be combined with a low pH value to provide high recovery values. The Pareto charts indicated the pH value as the most significant factor for CIPRO and MOXI (Figure 4 and Figure 12). These data also confirm the findings of LLE. pH is also the most limiting factor for the recovery of CIPRO and MOXI [12]. In the case of LEVO, pH, when analyzed in single, is not as significant as for CIPRO and MOXI. However, the interaction between pH and NaCl concentration is a significant factor (Figure 8).

### 4.4. The Optimization of Temperature

According to the literature data, the incubation temperature for CPE should be 15–20 °C above the cloud point temperature, which is 23 °C for TX-114 [2]. This is the temperature at which phase separation is observed and is characteristic of each surfactant. The temperature increase resulted in the phase’s dehydration and the disruption of the hydrogen bonds. It results in the enhancement of the preconcentration of the sample. The most commonly used temperatures are in the range of 40–60 °C. At higher temperatures, there is a risk of the decomposition of both the micelle and the analyte [1,2,4,5,17]. The lowest temperature analyzed in this study was 45 °C. At lower temperatures, the separation of the micelle-rich layer was not observed. According to the literature data, for TX-114, the used temperatures might be up to 60 °C [2]. For CIPRO, the impact of temperature was not significant for the TX-114 concentration range of 3–8%—the plateau was observed in the RSM diagram. The highest impact of temperature is observed for TX-114 concentrations below 3% (Figure 3). LEVO’s recovery increases when a high surfactant concentration is combined with a high temperature (Figure 7) and a low pH. In the case of MOXI, a lower temperature is preferable—too high a temperature leads to a decrease in recovery. It can be seen for concentrations of TX-114 lower than 3% (Figure 11). The analysis of the RSM graphs confirmed that the maximum recovery for CIPRO, LEVO, and MOXI is observed at temperatures of 45 °C, 60 °C, and 49 °C, respectively.

### 4.5. The Optimal Conditions

The highest recovery for LEVO is observed for a TX-114 concentration of 9% at pH 2.0 and a NaCl concentration of 4% at 60 °C. The optimal conditions for MOXI are a TX-114 concentration of 9%, pH 2.0, and NaCl 6.5% at a temperature of 49 °C. The theoretical recovery values are 74% and 81% for LEVO and MOXI, respectively. In the case of CIPRO, the conditions at which the highest recovery is observed are as follows: 5% TX-114, pH 2.0, and a temperature of 45 °C. The estimated recovery was 59%. In these conditions, the addition of salt was not necessary. The experimental measurements confirmed the estimated values for all analytes (Table 9). For CIPRO, in Figure 1, it can be seen that there is a second maximum observed at the high concentration of salt. The addition of salt results in a decrease in TX-114 concentration—the concentration of NaCl at 8% resulted in a decrease in TX-114 concentration, which should be 3.5%. pH and temperature remained at the same level. In these conditions, the estimated recovery should be 51%. The statistical analysis for the experiments conducted with and without adding neutral salt proved that the difference in the observed recovery for CIPRO (52% vs. 60%, respectively) was statistically significant.

The proposed optimal conditions for all analytes were validated experimentally. The data presented in Table 9 confirmed the repeatability of the procedure in the abovementioned conditions—the standard deviation did not exceed 2%. The observed values of recovery followed the theoretical data. They were tested for two levels of concentration: 1 and 8 mg/L. The observed experimental recoveries were identical to the theoretical values and did not depend on the analyte concentration—they were similar. Statistical analysis also showed no statistically significant differences between the analyzed concentrations for each analyte. The model’s validity for the optimized condition was also tested on human plasma. The recovery values obtained were similar to those obtained for the measurements performed on PBS.

The lowest value of recovery of CIPRO might be caused by the differences in their lipophilicity (logP). In the study of Blokhina et al. [18], LEVO was more lipophilic than CIPRO. In the study of Kłosińska-Szmurło et al., the logP value for LEVO is 0.74 and for MOXI is 0.96 [19]. Moreover, the results from the studies conducted by Czyrski et al. for CIPRO and LEVO and Langlois et al. for MOXI [20,21] confirmed that lipophilicity increased in the following order: CIPRO, LEVO, and MOXI. The analyzed compounds are lipophilic. However, the lowest logP value for CIPRO implies a lower permeability to the micelle. The analyzed fluoroquinolones are zwitterions. According to Cramariuc et al. [22], zwitterions may form the stacks in which the molecules’ antiparallel association is observed. That compensates for the electric field of the other molecule. That reduces the net potential. It results in easier penetration into the bilayer. This fact also explains the migration of the analyzed compounds into micelles.

## 5. Conclusions

CPE is a technique that reduces the use of organic solvents. Contrary to LLE, the extraction agent is a surfactant at a specific concentration. Such conditions must be found that provide the separation of the micelle-rich layer. The other factors that may impact the process are pH, the concentration of salt, and temperature. The concentration of TX-114 is the critical factor in the analysis of the recovery of LEVO with CPE. The other factor is the interaction of pH with the concentration of salt. In the case of CIPRO and MOXI, the predominant factor is pH. However, TX-114 concentrations also play an important role as limiting agents.

The analysis of the data obtained as a result of the experiments made it possible to determine the factors having the strongest influence on the recovery of CIPRO, LEVO, and MOXI and to determine the values of the factors allowing for the maximum recovery. This proves the applicability of CCD in the optimization process. The application of this model made it possible to quantitatively evaluate the impact of each of the independent variables on recovery for the cloud point extraction.

## Figures and Tables

**Table 1:** The design matrix for circumscribed CCD.

Experiment	TX-114 [%]	NaCl [%]	pH	Temperature [°C]
1	2	0	0	0
2	−1	1	1	1
3	−1	−1	−1	−1
4	−1	−1	−1	1
5	−1	1	1	−1
6	1	−1	1	1
7	0	0	−2	0
8	0	2	0	0
9	1	−1	1	−1
10	0	0	0	0
11	1	1	−1	1
12	1	1	−1	−1
13	0	0	2	0
14	−2	0	0	0
15	0	−2	0	0
16	−1	−1	1	−1
17	−1	−1	1	1
18	−1	1	−1	−1
19	−1	1	−1	1
20	1	−1	−1	−1
21	1	−1	−1	1
22	1	1	1	−1
23	1	1	1	1
24	0	0	0	−2
25	0	0	0	2
26	0	0	0	0
27	0	0	0	0

**Table 2:** The coded levels of the analysed independent variables.

Independent Variable	Level				
	−2	−1	0	1	2
TX-114 [%]	1.50	3.38	5.25	7.13	9.00
NaCl [%]	0.0	2.0	4.0	6.0	8.0
pH	2.0	4.0	6.0	8.0	10.0
Temperature [°C]	45.0	48.8	52.5	56.3	60.0

**Table 3:** The ANOVA^®^ analysis for CIPRO.

Independent Variables	MS	F	*p*
TX-114 [%] (L)	193.925	187.553	<0.000001
TX-114 [%] (Q)	226.013	218.587	<0.000001
NaCl [%] (L)	46.308	44.786	0.000022
NaCl [%] (Q)	40.681	39.344	0.000041
pH (L)	1201.962	1162.466	<0.000001
pH (Q)	0.018	0.017	0.897857
Temperature [°C] (L)	39.203	37.915	0.000049
Temperature [°C] (Q)	0.608	0.588	0.458012
TX-114 (L) × NaCl (L)	34.115	32.994	0.000092
TX-114 (L) × pH (L)	39.125	37.840	0.000049
TX-114 (L) × Temperature (L)	30.327	29.331	0.000156
NaCl (L) × pH (L)	8.470	8.192	0.014297
NaCl (L) × Temperature (L)	0.312	0.301	0.593142
pH (L) × Temperature (L)	16.732	16.182	0.001691

**Table 4:** The ANOVA^®^ analysis for LEVO.

Independent Variables	MS	F	*p*
TX-114 [%] (L)	2047.030	528.694	<0.000001
TX-114 [%] (Q)	33.472	8.645	0.012366
NaCl [%] (L)	384.881	99.404	<0.000001
NaCl [%] (Q)	56.847	14.682	0.002388
pH (L)	285.867	73.832	0.000002
pH (Q)	35.289	9.114	0.010683
Temperature [°C] (L)	158.569	40.954	0.000034
Temperature [°C] (Q)	104.066	26.878	0.000228
TX-114 (L) × NaCl (L)	29.133	7.524	0.017831
TX-114 (L) × pH (L)	11.072	2.860	0.116610
TX-114 (L) × Temperature (L)	0.815	0.210	0.654677
NaCl (L) × pH (L)	420.968	108.725	<0.000001
NaCl (L) × Temperature (L)	13.377	3.455	0.087749
pH (L) × Temperature (L)	192.308	49.668	0.000013

**Table 5:** The ANOVA^®^ analysis for MOXI.

Independent Variables	MS	F	*p*
TX-114 [%] (L)	984.577	306.505	<0.000001
TX-114 [%] (Q)	94.211	29.328	0.000156
NaCl [%] (L)	17.785	5.536	0.036514
NaCl [%] (Q)	77.132	24.012	0.000366
pH (L)	2985.970	929.552	<0.000001
pH (Q)	10.077	3.137	0.101901
Temperature [°C] (L)	40.352	12.562	0.004039
Temperature [°C] (Q)	4.348	1.354	0.267267
TX-114 (L) × NaCl (L)	2.295	0.714	0.414485
TX-114 (L) × pH (L)	80.103	24.936	0.000313
TX-114 (L) × Temperature (L)	20.160	6.276	0.027651
NaCl (L) × pH (L)	51.912	16.161	0.001699
NaCl (L) × Temperature (L)	5.593	1.741	0.211608
pH (L) × Temperature (L)	26.832	8.353	0.013572

**Table 6:** The validation parameters for CIPRO.

	Intraday		Interday	
Concentration [mg/L]	Precision [%]	Accuracy [%]	Precision [%]	Accuracy [%]
8	3.10	0.06	3.54	0.36
5	3.24	0.20	3.89	0.92
1	2.28	1.71	4.52	1.81
0.5	0.82	2.40	4.29	7.07

**Table 7:** The validation parameters for LEVO.

	Intraday		Interday	
Concentration [mg/L]	Precision [%]	Accuracy [%]	Precision [%]	Accuracy [%]
8	1.59	1.15	2.86	0.79
3	2.28	2.08	0.61	1.50
0.5	2.71	2.62	0.88	5.81
0.2	3.73	5.70	3.83	7.58

**Table 8:** The validation parameters for MOXI.

	Intraday		Interday	
Concentration [mg/L]	Precision [%]	Accuracy [%]	Precision [%]	Accuracy [%]
8	2.12	0.43	0.28	1.14
3	2.01	1.82	1.87	1.70
0.5	5.64	2.54	3.36	6.71
0.2	2.04	1.99	5.51	7.80

**Table 9:** The observed values of CIPRO, LEVO, and MOXI recoveries in the optimized CPE conditions.

Analyte	Concentration	
	1 mg/L	8 mg/L
CIPRO	60 ± 1.4%	60 ± 1.1%
LEVO	75 ± 1.8%	76 ± 0.7%
MOXI	84 ± 1.2%	84 ± 1.5%

## Data Availability

Not applicable.

## Supplementary Materials

The following supporting information can be downloaded at: https://www.mdpi.com/article/10.3390/pharmaceutics15061774/s1, Figure S1: The chromatogram for CIPRO analysis; Figure S2: The chromatogram for LEVO analysis; Figure S3: The chromatogram for MOXI analysis; Table S1: The results for the experiments for CIPRO; Table S2: The results for the experiments for LEVO; Table S3: The results for the experiments for MOXI.
