# Supplementary material for: The Chemometric Evaluation of the Factors Influencing Cloud Point Extraction for Fluoroquinolones
Source: Pharmaceutics. 2023 Jun 20;15(6):1774. doi: 10.3390/pharmaceutics15061774 (PMC10304411; doi:10.3390/pharmaceutics15061774)
Supplement: Supplementary file 1 [file pharmaceutics-15-01774-s001.zip › pharmaceutics-2445236-supplementary.pdf]

Supplementary information for '**The Chemometric Evaluation of the Factors Influencing Cloud Point Extraction for Fluoroquinolones'**

1. The chromatograms for the analyzed compounds.

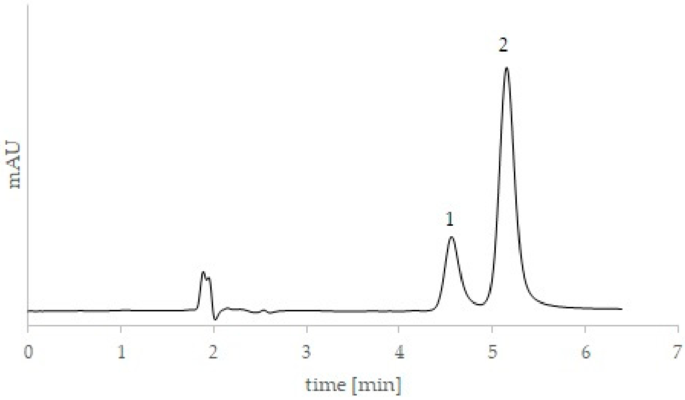

Figure S1. The chromatogram for CIPRO analysis (1 – internal standard, retention time: 4.6 min; 2 – CIPRO, retention time: 5.2 min). The peak's parameters for CIPRO –area under the peak – 109.2 [mAu*s]; symmetry – 1.1; tailing factor – 1.2; number of theoretical plates – 4379; resolution to the IS peak R_s – 1.94).

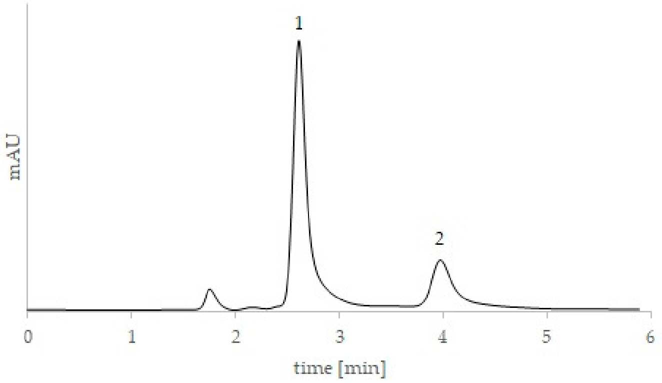

Figure S2. The chromatogram for LEVO analysis (1 – LEVO, retention time: 2.6 min; 2 – internal standard: retention time – 3.9 min). The peak's parameters for LEVO – area under the peak – 134.1 [mAU*s]; symmetry – 1.3; tailing factor – 1.4; number of theoretical plates – 2511; resolution to the IS peak R_s – 4.6).

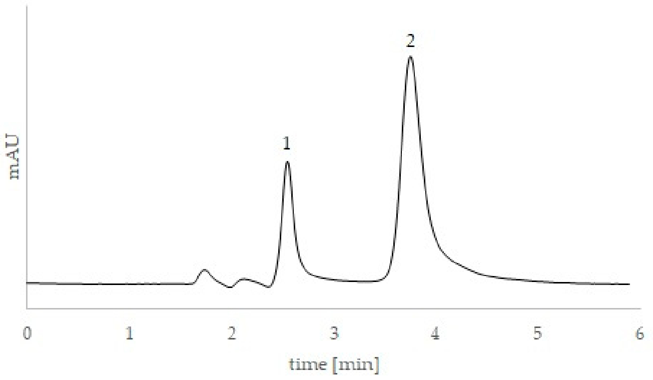

Figure S3. The chromatogram for MOXI analysis (1 – internal standard, retention time: 2.6 min; 2 – MOXI, retention time: 3.7 min). The peak's parameters for MOXI – area under the peak – 85.1 [mAU*s]; symmetry – 1.3; tailing factor – 1.4; number of theoretical plates – 2250; resolution to the IS peak R_s – 4.1).

The equations of linear correlation for the analyzed compounds are:

Equation for CIPRO: y = 0.6617x

Equation for LEVO: y = 0.2653x

Equation for MOXI: y=0.2169x

2. The results of the experiments for the analyzed compounds are:

Table S1. The results for the experiments for CIPRO.

TX-114	NaCl	pH	Temperature	Recovery [%]
2	0	0	0	20.88
-1	1	1	1	12.47
-1	-1	-1	-1	36.12
-1	-1	-1	1	28.79
-1	1	1	-1	16.60
1	-1	1	1	26.67
0	0	-2	0	40.52
0	2	0	0	30.78
1	-1	1	-1	22.49
0	0	0	0	27.64
1	1	-1	1	28.44
1	1	-1	-1	31.62
0	0	2	0	14.00
-2	0	0	0	7.84
0	-2	0	0	35.02
-1	-1	1	-1	14.91
-1	-1	1	1	11.35
-1	1	-1	-1	33.42
-1	1	-1	1	27.93
1	-1	-1	-1	38.21
1	-1	-1	1	36.54
1	1	1	-1	18.78
1	1	1	1	20.97
0	0	0	-2	30.98
0	0	0	2	25.13
0	0	0	0	26.30
0	0	0	0	27.57

Table S2. The results for the experiments for LEVO.

TX-114	NaCl	pH	Temperature	Recovery [%]
2	0	0	0	47.32
-1	1	1	1	9.27
-1	-1	-1	-1	28.82
-1	-1	-1	1	27.62
-1	1	1	-1	14.96
1	-1	1	1	39.49
0	0	-2	0	43.94
0	2	0	0	30.73
1	-1	1	-1	53.24
0	0	0	0	30.41
1	1	-1	1	51.48
1	1	-1	-1	47.07
0	0	2	0	29.76
-2	0	0	0	6.07
0	-2	0	0	45.74
-1	-1	1	-1	41.49
-1	-1	1	1	27.51
-1	1	-1	-1	27.23
-1	1	-1	1	30.14
1	-1	-1	-1	44.82
1	-1	-1	1	46.67
1	1	1	-1	38.75
1	1	1	1	24.67
0	0	0	-2	46.08
0	0	0	2	35.00
0	0	0	0	31.28
0	0	0	0	31.42

Table S3. The results for the experiments for MOXI.

TX-114	NaCl	pH	Temperature	Recovery [%]
2	0	0	0	43.55
-1	1	1	1	18.37
-1	-1	-1	-1	41.12
-1	-1	-1	1	33.23
-1	1	1	-1	19.51
1	-1	1	1	30.19
0	0	-2	0	64.97
0	2	0	0	36.76
1	-1	1	-1	31.62
0	0	0	0	40.27
1	1	-1	1	60.32
1	1	-1	-1	59.06
0	0	2	0	22.13
-2	0	0	0	21.24
0	-2	0	0	29.63
-1	-1	1	-1	23.55
-1	-1	1	1	22.71
-1	1	-1	-1	46.03
-1	1	-1	1	36.87
1	-1	-1	-1	57.47
1	-1	-1	1	52.85
1	1	1	-1	27.63
1	1	1	1	31.35
0	0	0	-2	41.75
0	0	0	2	36.24
0	0	0	0	41.20
0	0	0	0	40.85

3. The full equations for recovery for the analysed compounds:

$Recovery_{CIPRO}$ = 27.170 + 2.846 × TX-114 – 3.255 × $(TX\text{-}114)^2$ – 1.389 × NaCl + 1.381 × $(NaCl)^2$ – 7.077 × pH – 0.029 × pH^2 – 1.278 × temperature + 0.168 × $(temperature)^2$ – 1.460 × TX-114 × NaCl + 1.564 × TX-114 × pH + 1.377 TX-114 × temperature + 0.728 × NaCl × pH - 0.139 × NaCl × temperature + 1.022 × pH × temperature

$Recovery_{LEVO}$ = 31.037 + 9.235 × TX-114 – 1.253 × $(TX\text{-}114)^2$ - 4.005 × NaCl + 1.632 × $(NaCl)^2$ – 3.451 × pH + 1.286 × pH^2 – 2.570 × temperature + 2.209 × $(temperature)^2$ + 1.349 × TX-114 × NaCl – 0.832 × TX-114 × pH – 0.226 TX-114 × temperature – 5.129 × NaCl × pH + 0.914 × NaCl × temperature – 3.467 × pH × temperature

$$Recovery_{MOXI} = 40.773 + 6.405 \times TX\text{-}114 - 2.101 \times (TX\text{-}114)^2 + 0.861 \times NaCl - 1.901 \times (NaCl)^2 - 11.154 \times pH + 0.687 \times pH^2 - 1.297 \times temperature - 0.451 \times (temperature)^2 + 0.379 \times TX\text{-}114 \times NaCl - 2.238 \times TX\text{-}114 \times pH + 1.1225\ TX\text{-}114 \times temperature - 1.801 \times NaCl \times pH + 0.591 \times NaCl \times temperature + 1.295 \times pH \times temperature$$
